# Hybrid Functional ITO/Silver Nanowire Transparent Conductive Electrodes for Enhanced Output Efficiency of Ultraviolet GaN-Based Light-Emitting Diodes

**DOI:** 10.3390/ma17215385

**Published:** 2024-11-04

**Authors:** Munsik Oh, Mun Seok Jeong, Jaehee Cho, Hyunsoo Kim

**Affiliations:** 1 School of Semiconductor and Chemical Engineering, Jeonbuk National University, Jeonju 54896, Republic of Korea; yohan.lee@skkeyfoundry.com; 2 Department of Semiconductor Science and Technology, Jeonbuk National University, Jeonju 54896, Republic of Korea; 3 Department of Physics, Department of Energy Engineering, Hanyang University, Seoul 04763, Republic of Korea; mjeong@hanyang.ac.kr

**Keywords:** silver nanowire, localized surface plasmon, ultraviolet light-emitting diodes

## Abstract

We investigated hybrid functional transparent conductive electrodes (HFTCEs) composed of indium-tin-oxide (ITO) and silver nanowires (AgNWs) for the enhancement of output efficiency in GaN-based ultraviolet light-emitting diodes (UVLEDs). The HFTCEs demonstrated an optical transmittance of 69.5% at a wavelength of 380 nm and a sheet resistance of 16.4 Ω/sq, while the reference ITO TCE exhibited a transmittance of 76.4% and a sheet resistance of 18.7 Ω/sq. Despite the 8.9% lower optical transmittance, the UVLEDs fabricated with HFTCEs achieved a 25% increase in output efficiency compared to reference UVLEDs. This improvement is attributed to the HFTCE’s twofold longer current spreading length under operating forward voltages, and more significantly, the enhanced out-coupling of localized surface plasmon (LSP) resonance with the trapped wave-guided light modes.

## 1. Introduction

For the fabrication of high-efficiency GaN-based light-emitting diodes, it is essential to employ high-quality transparent conductive electrodes (TCEs) that exhibit superior optical transmittance, low sheet resistance, and form excellent Ohmic contact with the p-type cladding layer [1,2]. Currently, indium-tin-oxide (ITO) is widely used as the p-type electrode because it meets these critical requirements [3,4,5]. For example, ITO has been reported to form good Ohmic contact on p-GaN with a typical specific contact resistance of 10^−3^−10^−4^ Ω cm^2^, a sheet resistance (*R*_sh_) of 10–30 Ω/sq. and an optical transmittance higher than 90% in the visible wavelength range. However, for UVLEDs emitting at wavelengths below 400 nm, the use of ITO becomes challenging, as its optical transmittance drops significantly in this range, leading to substantial efficiency losses [3,4,5].

To address this issue, various novel TCEs incorporating nanomaterials or functional structures have been investigated [6,7,8,9,10,11,12,13,14,15,16,17,18,19]. Specifically, carbon nanotubes have been explored as TCEs for LEDs, where their optical transmittance exceeding 85% in the visible and UV wavelength ranges is a major advantage [6,7]. However, the relatively poor *R*_sh_ value (~300 Ω/sq.) and the difficulty in achieving Ohmic contact with p-GaN remain unresolved [6,7]. Graphene has also been extensively investigated due to its good optical transmittance in the UV wavelength region, as well as its acceptable *R*_sh_ values, and relatively high potential for mass production [8,9,10,11]. However, the large work function mismatch between graphene (4.2–4.5 eV) [12] and p-GaN (~7.5 eV) [1,2] prevents the formation of Ohmic contact, hindering its practical use in UVLEDs. In response to these challenges, hybrid structures such as graphene/ITO nanodots [13], ITO/graphene/ITO [14], ITO/Au/ITO [15], zinc gallate/ITO [16], and Ni/Au metal meshes [17] have been studied to reduce *R*_sh_ (or enhance current spreading) and increase optical transmittance in the UV wavelength region.

Recently, silver nanowires (AgNWs) have also been introduced as TCEs for UVLEDs due to their good optical transmittance and low *R*_sh_ values [20,21,22]. However, the main issues with AgNWs include poor Ohmic contact and relatively unstable or less reproducible formation of AgNW networks, which are typically associated with the solution-based process used for AgNW coating [23]. This indicates that a supporting layer for Ohmic contact and/or an additional layer for current spreading should be employed for practical applications. In this regard, Seo et al. [24] used AgNWs/graphene hybrid TCEs for UVLEDs, while Park et al. employed ITO nanodots [25] or thin ITO films [26] as an Ohmic contact layer combined with overlying AgNWs, achieving significant output enhancements of 24–62% compared to reference UVLEDs.

In this study, we developed hybrid functional TCEs (HFTCEs) composed of ITO and AgNWs for UVLEDs. In the HFTCEs, the underlying ITO layer serves as the Ohmic contact layer and current spreader, while the overlying AgNWs primarily function as a current spreader and vertical light out-coupler via localized surface plasmon (LSP) resonance [27,28]. Notably, unlike similar structures proposed by other groups [25,26], our HFTCEs feature a sufficiently thick ITO contact layer (100 nm) to ensure excellent Ohmic contact and substantial current spreading, despite some degradation in optical transmittance. The reduced transmittance is compensated by the functional properties of the overlying AgNWs, which enhance light extraction through LSP resonance.

## 2. Materials and Methods

**Figure 1:**
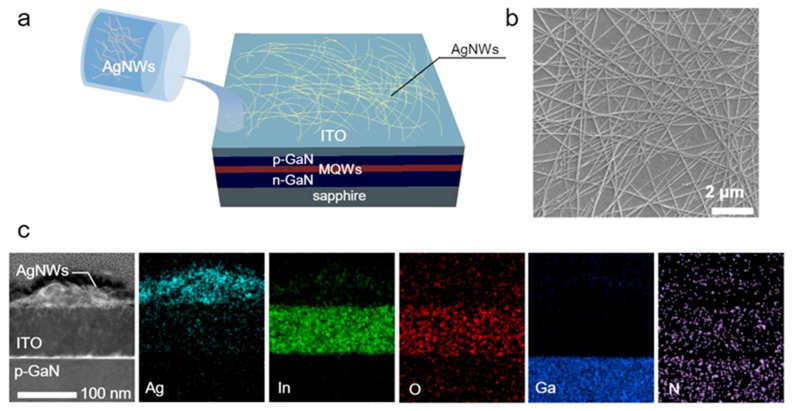
(**a**) The schematic diagram of process and (**b**) SEM top-view images and (**c**) cross-sectional STEM and EDXS elementary maps of ITO/AgNWs HFTCE.

To form ITO/AgNW HFTCEs, a 100 nm-thick ITO film was first deposited using an e-beam evaporator on double-side polished sapphire substrates (for TCE evaluation) or on the top p-GaN layer of UVLEDs (for LED fabrication). The films then underwent rapid thermal annealing (RTA) at 550°C for 1 min in air. For the AgNW coating, the as-received AgNW dispersion (Cambrios ClearOhm Ink) was sonicated for 300 s, followed by spin-coating onto the ITO films for 40 s at 800 rpm. The process flow and scanning transmission electron microscopy (STEM) top-view images of the HFTCE are shown in Figure 1a,b. The cross-sectional views of the HFTCEs, analyzed using STEM and energy-dispersive X-ray spectroscopy (EDXS), are displayed in Figure 1c. The AgNWs were evenly distributed over the underlying ITO layer and exhibited good interconnections, ensuring efficient electrical contact. The optical transmittance and sheet resistance (*R*_sh_) of the TCEs (prepared on sapphire substrates) were measured using a UV/VIS spectrometer (V-670EX) and a four-point probe system (CMT-SR1000N). To evaluate the contact properties, a transmission line model (TLM) method was employed, with contact pads sized 150 μm × 200 μm and gap spacings of 10, 20, 40, and 60 μm. The electrical characteristics of contacts were measured using a parameter analyzer (HP4156A).

For UVLED fabrication, rectangular mesas were dry-etched to a depth of ~1.0 μm using an inductively coupled plasma etching system. Ti/Al/Ni/Au (30/70/30/70 nm) was then deposited on the exposed n-GaN as the n-electrode via e-beam evaporation, followed by RTA at 550 °C for 1 min in nitrogen ambient. To form TCEs on the p-GaN layer, the ITO film was deposited on the mesa and annealed using the same RTA process. A Ti/Au (20/10 nm) probing pad was subsequently deposited on the TCEs. AgNWs were selectively spin-coated on the top ITO via a lift-off technique [19], with spin-coating performed in the final step to minimize contamination of the AgNWs by the photolithographic process. The UVLED wafer used in this study consisted of ~3.0 μm of undoped GaN, 3.5 μm of n-GaN, multiple quantum wells (MQWs) with an emission peak at ~380 nm, a p-AlGaN-based electron blocking layer, and a ~0.15 μm p-GaN layer.

The fabricated UVLEDs were evaluated using a parameter analyzer connected to a photodiode (UV-818) positioned approximately 1 cm above the LED chips. Spatially-resolved electroluminescence (EL) images were acquired using a confocal scanning electroluminescence microscope (CSEM) [29,30]. Time-resolved photoluminescence (TRPL) measurements were conducted using a multifunctional confocal microscope with time-correlated single photon counting (TCSPC) (NTEGRA, NT-MDT). A 405 nm pulsed laser with a repetition rate of 80 MHz and a pulse width of 80 ps was used as the excitation source.

## 3. Results and Discussion

**Figure 2:**
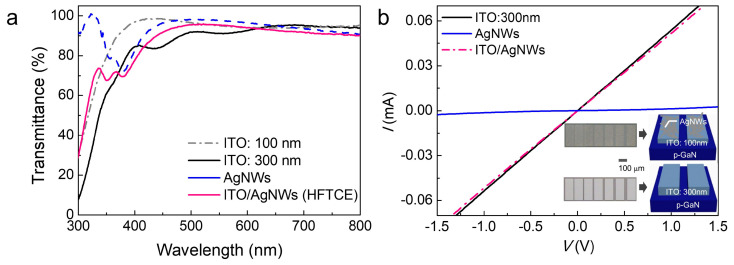
(**a**) The optical specular transmittance spectra of the ITO films (100 nm and 300 nm), AgNWs, and ITO (100 nm)/AgNWs HFTCE. (**b**) *I–V* curves of the reference 300 nm thick ITO, AgNWs, and ITO/AgNWs TLM patterns formed on the p-type layer of UVLEDs. The insets show the optical microscopic top-view images and schematic diagrams of TLM patterns for AgNWs and ITO/AgNWs.

Figure 2a presents the optical specular transmittance spectra of the ITO films (100 nm and 300 nm), AgNWs, and ITO (100 nm)/AgNWs HFTCEs. The transmittance of the 300 nm thick ITO film exhibited fluctuations, attributed to the interference effects arising from film thickness fringes. Notably, ITO films exhibited high transmittance in the visible and infrared ranges but experienced a significant drop below 400 nm due to band-to-band absorption. Consequently, the optical transmittance at 380 nm was 93.1% for the 100 nm thick ITO film and 76.4% for the 300 nm thick ITO film. In contrast, AgNWs showed high transmittance over 300–800 nm, except for a sharp decline around ~380 nm caused by transverse surface plasmon resonance [31]. The transmittance of AgNWs at 380 nm was 72.2%. As a result, the ITO/AgNWs HFTCE exhibited the relatively low transmittance value of 69.6%, which is 8.9% lower than that of the 300 nm thick ITO TCE (hereinafter referred to as the reference TCE). Indeed, the transmittance of the ITO/AgNWs HFTCE is much lower than the mean value (82.6%) of the 100 nm thick ITO and AgNWs. We suspect two primary factors for this result: first, the reproducibility issues of the ITO or AgNWs films, and second, the sensitive change in the transmittance of the AgNWs around 380 nm due to surface plasmon resonance. This may indicate reproducibility issues not only from the material perspective but also in the transmittance measurements. Nonetheless, we proceeded with the experiments to report the findings.

On the other hand, the HFTCE demonstrated better *R*_sh_ values compared to the reference TCE, with *R*_sh_ values of 18.7 Ω/sq for the 300 nm thick ITO, 63.6 Ω/sq for the 100 nm thick ITO, 19.9 Ω/sq for the AgNWs, and 16.4 Ω/sq for the ITO/AgNWs HFTCE. Based on these data, we first selected the 300 nm thick ITO films as the reference TCEs due to their relatively high optical transmittance (76.4%) and reasonably low *R*_sh_ of 18.7 Ω/sq. Second, it is meaningful to discuss the *R*_sh_ value of the HFTCE. The *R*_sh_ of HFTCE can be estimated using a parallel resistance model, expressed as 1/*R*_sh_ = 1/*R*_sh,ITO_ + 1/*R*_sh,AgNWs_, which yields an *R*_sh_ of 15.2 Ω/sq. It is noteworthy that the measured *R*_sh_ value of 16.4 Ω/sq is in good agreement with the calculated value. This indicates that the parallel resistance model is a good approximation for explaining the carrier transport mechanism of ITO/AgNWs.

Generally, the parallel resistance model assumes that there is no contact resistance between the two layers, indicating that the contact resistance between ITO and AgNWs is not significant. Additionally, several factors need to be considered for measurements of the *R*_sh_ of ITO/AgNWs. Firstly, the AgNWs are entangled, and due to their geometrical characteristics, there is a possibility of increased resistance from imperfect contact with the probing tip, which may result in measured *R*_sh_ values being higher than the theoretical value. Secondly, during *R*_sh_ measurements using four-point probing, there is a possibility of contact between the probing tip and ITO. In such cases, the probing tip could make contact with both the AgNWs and ITO simultaneously, allowing current to flow, which may limit the strict application of the parallel resistance model. Nonetheless, since the actual measurement is nearly identical to the theoretical value, it is important to understand carrier transport simply using the parallel resistance model.

Figure 2b shows the current–voltage (*I–V*) curves of the reference 300 nm thick ITO, AgNWs, and ITO/AgNWs TLM patterns formed on the p-type layer of UVLEDs. The *I–V* curves were measured between adjacent contact pads with a spacing of 10 μm, as illustrated in the inset. The inset also shows the optical microscopic top-view images and schematic diagrams of the TLM patterns for AgNWs and ITO/AgNWs. It is clearly shown that the *I–V* curves of the ITO and ITO/AgNWs were nearly identical and quite linear, indicating that Ohmic contacts were formed for both the reference and hybrid TCEs. The specific contact resistance was 7.3 × 10^−4^ Ω·cm^2^ for the ITO and 7.9 × 10^−4^ Ω·cm^2^ for the ITO/AgNWs. Indeed, this value is sufficiently low for use in practical devices [1]. In contrast, the AgNWs exhibited the poorest *I–V* performance, with a contact resistance of 1.1 × 10⁻^2^ Ω·cm². This could be attributed to poorer electrical connections between the AgNWs and the probe or worse contact between the AgNWs and p-GaN. Indeed, this is the rationale behind introducing the ITO/AgNW HFTCE instead of using AgNWs alone. In addition, it is noteworthy that the TLM evaluation of AgNW contacts may not be fully accurate due to issues with poor electrical contact. Therefore, for precise evaluation, specifically designed TLM patterns and processes should be used, as reported in the literature [21]. However, obtaining the exact contact values of AgNWs is not the main focus of this study.

**Figure 3:**
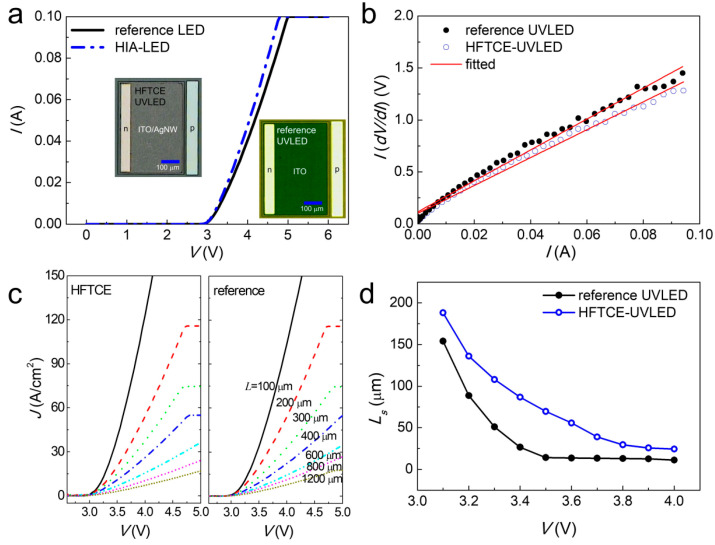
(**a**) *I*–*V* curves, (**b**) *I*(d*V*/d*I*) verus *I* plots, (**c**) *J*–*V* curves plotted as a function of mesa length (*L*), and (**d**) *L*_s_ versus forward voltage plots of HFTCE-UVLEDs and reference UVLEDs. The optical microscopic top views of both UVLEDs are shown in the inset of (**a**).

Figure 3a shows the *I–V* curves of UVLEDs fabricated with the reference ITO (reference UVLED) and the HFTCEs (HFTCE-UVLED). The optical microscopic top views of both UVLEDs are displayed in the inset of Figure 3a. It is noteworthy that the HFTCE-UVLED exhibited a steeper *I–V* curve compared to the reference UVLED. For example, the turn-on voltage measured at 20 mA was 3.51 V for the HFTCE-UVLED and 3.59 V for the reference UVLED. According to the literature [32], the turn-on voltages (*V*_on_) having a QW active layer can be obtained using the equation: *V*_on_ = *E*_g_/*e* + *IR*_s_ + (Δ*E*_C_ − *E*_0,*e*_)/*e* + (Δ*E*_V_ − *E*_0,h_)/*e*, where *E*_g_ is the bandgap energy, *e* is the electronic charge, *R*_s_ is the series resistance, Δ*E*_C_ (Δ*E*_V_) is the conduction (valence) band offsets, and *E*_0,*e*_ (*E*_0,h_) is the ground state energy of the electron (hole) in the QW. Considering that the first, third, and fourth terms of the equation are identical for both samples (since the same UVLED wafers were used), the change in *V*_on_ can be directly attributed to the change in *R*_s_.

To obtain *R*_s_ values, *I–V* data were replotted to *I*(d*V*/d*I*) verus *I* as shown in Figure 3b, where the *R*_s_ can be obtained from theoretical fitting according to the equation: *I*(d*V*/d*I*) = *R*_s_*I* + *nkT*/*e*. Here, *n* is the ideality factor, *k* is the Boltzmann constant, and *T* is the absolute temperature. Consistently, the *R*_s_ was found to be 13.4 Ω for the HFTCE-UVLED and 15.1 Ω for the reference UVLED.

Considering that the Ohmic properties were nearly the same for both UVLEDs, the improved electrical performance of the HFTCE-UVLEDs can be attributed to the lower *R*_sh_ values of the ITO/AgNWs compared to the reference ITO. To further investigate the effect of *R*_sh_ on the electrical properties of the UVLEDs, the current density versus voltage (*J–V*) curves were plotted as a function of mesa length (*L*), as shown in Figure 3c. Based on the measured *J*–*V* curves and *J*–*L* plots the current spreading length (*L*_s_), defined as the length over which the current density (*J*) drops to the 1/*e* value at the mesa edge, could be obtained according to *J*(*L*) = *J*_0_(*L*/*L*_s_) − 1[1-exp(−*L*/*L*_s_)], where *J_0_* is the current density at the mesa edge (see Figure 3d) [33].

In Figure 3d, it is clearly shown that the *L*_s_ of the HFTCE-UVLED is longer than that of the reference UVLED. For example, the *L*_s_ ranges from approximately 56 to 136 µm for the HFTCE-UVLED, while it ranges from 14 to 89 µm for the reference UVLED at forward voltages of 3.2 to 3.6 V. It is noteworthy that the longer *L*_s_ can increase the effective active area of device, thereby reducing Joule heating and mitigating efficiency droop [34,35].

**Figure 4:**
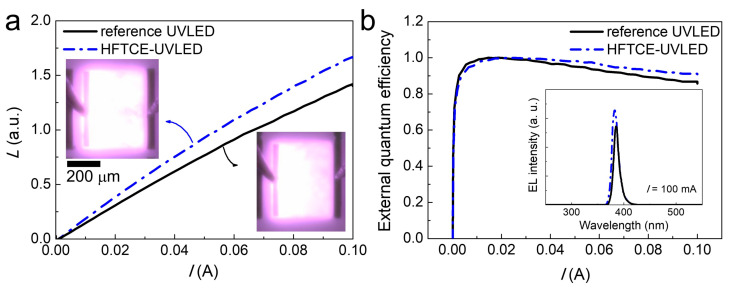
(**a**) *L–I* curves and (**b**) normalized external quantum efficiency versus *I* plots HFTCE-UVLEDs and reference UVLEDs. The EL images of the UVLEDs taken at an injection current of 10 mA and the EL spectra are shown in the insets of (**a**,**b**).

Figure 4a shows the light output power versus injection current (*L–I*) plots for both UVLEDs. The electroluminescence (EL) images of the UVLEDs taken at an injection current of 10 mA are also shown in the insets of Figure 4a. It is evident that the *L–I* curve of the HFTCE-UVLED was significantly improved compared to that of the reference UVLED; specifically, the light output (*L*) of the HFTCE-UVLED at an injection current of 20 mA was 25% higher than that of the reference UVLED. Consistently, the EL images of the HFTCE-UVLED also showed brighter and more uniform light emission compared to the reference UVLED. One of the major challenges in the output performance of UVLEDs is minimizing output degradation with increasing current, a phenomenon known as efficiency droop. To investigate whether the HFTCE improved the efficiency droop problem, the *L–I* curves were replotted to show external quantum efficiency versus current, as shown in Figure 4b.

In Figure 4b, it is clearly shown that the HFTCE-UVLED exhibited smaller efficiency droop compared to the reference UVLED, where the efficiency droop was defined herein as the relative drop in efficiency from its peak value to that at *I* = 100 mA. Specifically, the efficiency droop was only 11.4% for the HFTCE-UVLED, while it was 16.4% for the reference UVLED. Considering that efficiency droop is primarily a function of current density and temperature [34,35,36], this finding indicates that the HFTCE played a crucial role in reducing efficiency droop by increasing the effective active area of the devices (or increasing *L*_s_). This conclusion is further supported by the inspection of EL spectra taken at 100 mA, as shown in the inset of Figure 4b. Specifically, the HFTCE-UVLED exhibited a peak wavelength of 382.4 nm, which is 2.5 nm blue-shifted compared to the reference UVLED at 384.9 nm. This suggests that the reduced heating effect, resulting from decreased device series resistance (or an increased effective active area), may contribute to the blue shift in the EL spectrum.

One of the most competitive TCEs in GaN-based UVLEDs, as reported by other groups, is the ITO nanodots/AgNWs or thin ITO/AgNWs TCE structures [25,26], as mentioned in the introduction section. The core idea of these two structures is to minimize optical absorption loss in ITO by utilizing either the ITO nanodots structure or a thin ITO layer (10 nm) as the Ohmic contact layer. This approach allows for minimizing the reduction in transmittance. However, in actual UVLED applications, critical drawbacks emerge due to the incomplete Ohmic contact of ITO and/or the reliance on the AgNWs layer for current spreading without assistance from ITO. This results in an increase of *V*_on_ or *R*_s_. For example, in the case of the ITO nanodots/AgNWs TCE, while the *L* was improved by 24–62% compared to the reference UVLED, the *V*_on_ increased from 3.5 V to 3.52–3.75 V. Similarly, for the thin ITO/AgNWs structure, the *L* was enhanced by 7.9–14.0% compared to the reference UVLED, but the *V*_on_ increased from 3.45 V to 3.5–3.55 V, and the *R*_s_ increased from 13.2 Ω to 16.8–18.0 Ω. In contrast, our ITO/AgNWs HFTCE demonstrated a significant advantage by applying a thicker ITO layer, which not only improves the *V*_on_ but also enhances the *L* as a result of better current spreading.

Until now, the primary reasons for the improvements in the electrical and optical output characteristics of the HFTCE-UVLED have been attributed to the enhanced current spreading effect. While this explanation for the improvement in the electrical properties of the HFTCE-UVLED seems to be reasonable, it remains questionable whether the alleviated current crowding effect can account for the 25% increase in optical output power. Furthermore, it is important to note that the ITO/AgNWs HFTCE exhibited 8.9% lower optical transmittance compared to the reference ITO TCE.

**Figure 5:**
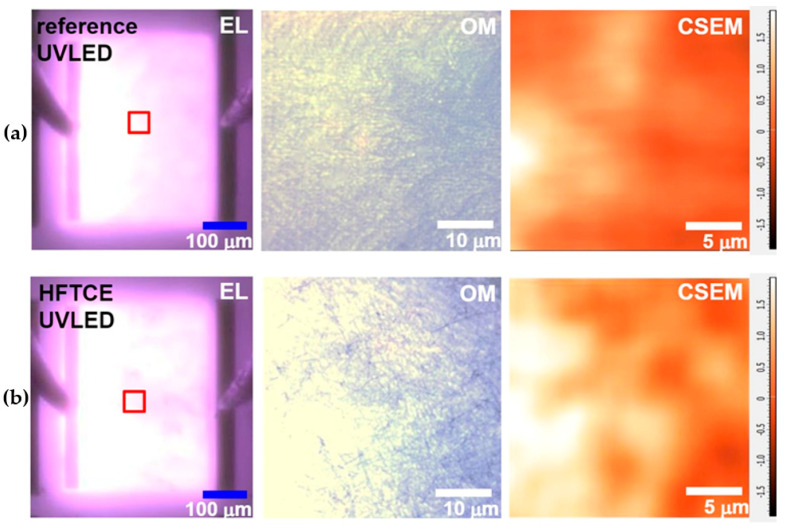
The EL, magnified OM, and CSEM images of (**a**) reference UVLED and (**b**) HFTCE-UVLED taken at 8 mA.

To clarify the origin of the output enhancement, magnified OM and spatially resolved EL images were obtained using a CSEM at an injection current of 8 mA (see Figure 5). The targeted areas of the devices for these measurements are denoted by red rectangles in the EL images. Inspection of the EL images reveals that, in the reference UVLED, current crowds at the mesa edges near the n-electrode (left side), whereas the current distribution is relatively more uniform in the HFTCE-UVLED, consistent with the obtained *L*_s_ data. Notably, the magnified OM images revealed that light emission was significantly brighter at several localized spots in the HFTCE-UVLED. At similar positions, the CSEM image of the HFTCE-UVLED also showed very bright local zones at specific locations. In contrast, the reference UVLED showed fewer bright local zones. According to our previous study [21], the observed emission inhomogeneity was attributed to either inhomogeneous AgNW contact with p-GaN or poor electrical interconnection among the densely packed AgNWs. However, this explanation appears less applicable in the current study, as the use of an underlying ITO contact should alleviate issues related to Ohmic contact and current spreading. Therefore, it is likely that the observed inhomogeneous bright local zones are associated with the vertical out-coupling of guided modes.

**Figure 6:**
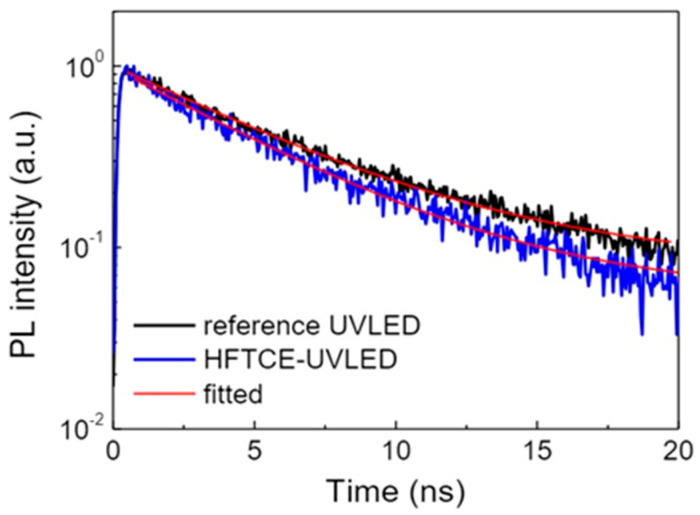
PL—intensity decay curves of HFTCE-UVLEDs and reference UVLEDs.

To investigate this hypothesis, PL intensity decay curves were obtained for both UVLEDs using TRPL measurements, as shown in Figure 6. This figure clearly shows that the PL intensity of the HFTCE-UVLED decayed more rapidly than that of the reference UVLED. The decay curves was fitted using a monoexponential decay, i.e., PL intensity ~ exp(*t*/τ), where *t* is the time and τ is the PL decay time constant corresponding to exciton lifetime. For example, the exciton lifetimes were found to be 5.6 ns for the reference UVLED and 4.8 ns for the HFTCE-UVLED. It is noteworthy that, in previous studies involving systems with Ag nanostructures, the decrease in exciton lifetime has been attributed to the coupling of the QWs with LSP from the Ag nanoparticles, leading to a significant enhancement in light output [27,28,37,38].

It is worthwhile to consider whether LSP resonance can occur despite the relatively large distance (~250 nm) between the AgNWs and the QW active region. Specifically, the penetration depth of the surface plasmon fringing field into the GaN has been reported to be approximately 40 nm at blue and UV wavelengths [28], which is considerably shorter than the physical distance of 250 nm. This suggests that a strong LSP mode is unlikely to occur under these conditions.

**Figure 7:**
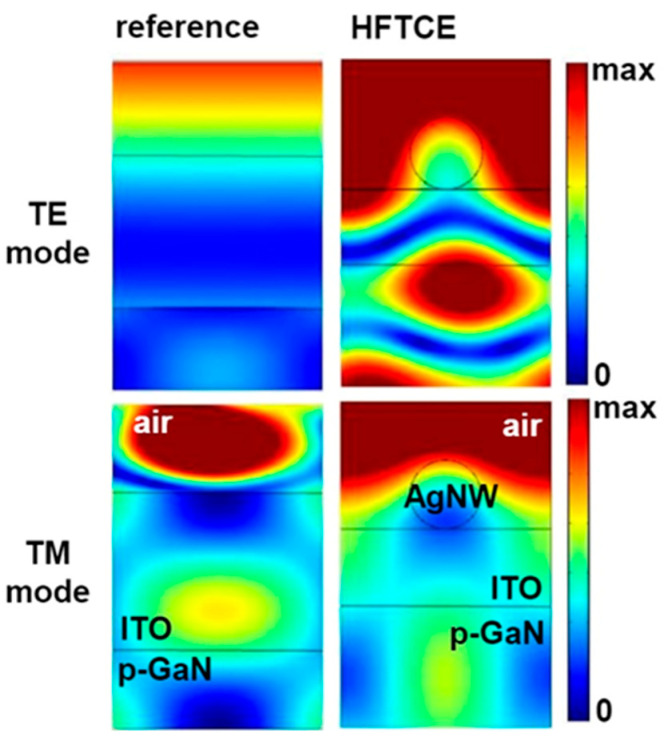
Electric field distributions at the cross-section of HFTCE-UVLEDs and reference UVLEDs, computed for TE and TM mode.

To address this issue, the electric field distributions at the cross-section of the reference UVLED and the ITO/AgNWs HFTCE-UVLEDs were computed for both transverse electric (TE) and transverse magnetic (TM) modes using commercial finite element-based software (Comsol Multiphysics with RF module) (see Figure 7). In this simulation, both configurations maintained the same device geometry, with the p-GaN and ITO layers each having a thickness of 250 nm, and AgNWs with a diameter of 30 nm positioned on the ITO layer. The 380 nm light source was located within the GaN active region. In the TE mode, the reference structure displayed a relatively uniform field distribution, with gradual transitions across the layers, indicating minimal field localization. In contrast, the HFTCE structure showed significant field enhancement in the TE mode, particularly above the p-GaN layer. For the TM mode, the reference structure exhibited limited field concentration at the air/ITO interface, likely due to the absence of LSP resonance, resulting in weaker field confinement. However, the HFTCE structure with AgNWs demonstrated strong field localization near the AgNW interface, attributed to the high reflectivity of silver, which facilitates LSP resonance and amplifies the field near the AgNWs surface. This pronounced field intensification in the TM mode indicates the potential of the HFTCE structure for applications in UVLEDs, where LSP resonance can enhance light extraction efficiency.

According to the literature [28], it has been demonstrated that AgNWs with dimensions smaller than the wavelength of light can achieve LSP resonance under specific conditions, thereby enhancing the electric field in the vicinity of the LSP and significantly affecting the spontaneous light emission of the QWs in LEDs. Under these conditions, energy transfer from the excitons in the QWs to the LSP can occur, leading to the formation of effective light out-coupling channels and improving light extraction efficiency. Consequently, the much stronger localized electric field observed at the cross-section of the HFTCE-UVLED suggests a significantly higher probability of light output coupling, which agrees well with the experimental findings.

Consequently, the enhanced output power of the HFTCE-UVLED is primarily attributed to the functional characteristics of the ITO/AgNWs, which significantly increase extraction efficiency through LSP resonance with the trapped wave-guided modes of light. This explanation is more reasonable, considering that, as mentioned earlier, the slight improvement in *R*_sh_ with degraded transmittance using HFTCE is insufficient to account for the 25% output enhancement. On the one hand, contrary to the predicted penetration depth of the surface plasmon fringing field into GaN (approximately 40 nm), the reason for the strong localized electric fringing field observed near the AgNWs in simulations and experiments, despite the much longer distance (250 nm) between the AgNWs and the QWs, remains unclear and is currently under investigation.

## 4. Conclusions

To summarize, the ITO/AgNWs HFTCE was employed in GaN-based UVLEDs to improve both electrical and optical output characteristics. The electrical properties of the HFTCE-UVLED showed significant improvement, which can be attributed to the reduced *R*_sh_ of the HFTCE, leading to increased *L*_s_ or effective active area. Notably, despite the much lower optical transmittance of the ITO/AgNWs HFTCE (69.5%) compared to the reference ITO TCE (76.4%), the HFTCE-UVLED demonstrated a remarkable 25% increase in EL emission brightness over the reference UVLED. This enhancement can primarily be attributed to the functional characteristics of the ITO/AgNWs, which dramatically improve light extraction efficiency through LSP resonance with the trapped wave-guided modes of light.

## Data Availability

Data are contained within the article.
